# Trends in Healthcare-Acquired Infections Due to Multidrug-Resistant Organisms at a German University Medical Center Before and During the COVID-19 Pandemic

**DOI:** 10.3390/microorganisms13020274

**Published:** 2025-01-25

**Authors:** Susanne Kolbe-Busch, Paule Dana Djouela Djoulako, Catalina-Suzana Stingu

**Affiliations:** 1 Institute of Hygiene, Hospital Epidemiology and Environmental Medicine, University of Leipzig Medical Center, 04103 Leipzig, Germany; susanne.kolbe-busch@medizin.uni-leipzig.de (S.K.-B.); danakamga2019@yahoo.com (P.D.D.D.); 2 Institut Pasteur/Cnam (Conservatoire National des Arts et Métiers), 75015 Paris, France; 3 Institute for Medical Microbiology and Virology, University of Leipzig Medical Center, 04103 Leipzig, Germany

**Keywords:** MDRO, healthcare-associated infections, inpatients, COVID-19

## Abstract

Background: Healthcare-acquired infections due to multidrug-resistant organisms (MDR-HAIs) pose globally significant challenges to healthcare systems, leading to increased morbidity, mortality, and healthcare costs. According to the World Health Organization, the COVID-19 pandemic significantly impacted the burden of MDR-HAIs. The aim of this study was to investigate the dynamics and epidemiology of MDR-HAIs in inpatients at the University of Leipzig Medical Center (ULMC) before and during the COVID-19 pandemic. Methods: We compared data from inpatients with bacterial infections from 2017 to 2019 (pre-COVID-19) and from 2021to 2023 (during COVID-19) in a cross-sectional, monocentric, retrospective survey. This study focused on selected multidrug-resistant organisms (MDROs) and four clinical specimens. We analyzed the risk factors for MDR-HAIs using logistic regression models. Results: Out of 342,705 inpatients, 32,206 were diagnosed with a bacterial infection. The prevalence increased significantly from 8.09% (pre-COVID-19) to 10.79% (during COVID-19) (*p* < 0.001), but the proportion of MDROs decreased from 10.14% to 8.07%. The proportions of MDR-HAIs were 59.86% and 56.67%, respectively. The proportion of carbapenem-resistant *Klebsiella pneumoniae* and *Enterobacter cloacae* increased significantly. The risk of MDR-HAIs during COVID-19 decreased by 18% compared to pre-COVID-19 (*p* = 0.047). Longer hospital stays increased the risk of MDR-HAIs in both periods. This risk significantly decreased for children and the elderly during COVID-19. Conclusion: These findings show that it is also important to analyze epidemiological data at the facility level in order to evaluate the effectiveness of infection control practices even during unprecedented health crises like the COVID-19 pandemic.

## 1. Introduction

Nosocomial infections, also known as hospital-acquired infections (HAIs), as defined for in-hospital surveillance by the Centers for Disease Control and Prevention’s (CDC’s) National Healthcare Safety Network, refer to infections that are not present on admission and manifest later than 2 days after hospital admission [1]. They are a significant public health concern, particularly due to the rise of multidrug-resistant organisms (MDROs) [2]. These infections occur in healthcare settings and are often difficult to treat because the pathogens involved are resistant to multiple classes of antimicrobials [3]. MDR-HAIs are associated with increased morbidity, mortality, and healthcare costs [4]. According to the CDC, approximately 1 in 31 hospitalized patients develops at least one HAI each day [5]. The global burden of MDROs is alarming, with studies estimating that infections caused by these organisms could result in 10 million deaths annually by 2050 if current trends continue [6]. The World Health Organization (WHO) also highlights the global burden of HAIs, emphasizing their prevalence in intensive care units and their impact on patient outcome [7].

The acquisition of MDROs in healthcare settings is a complex process influenced by many factors, such as direct contact with contaminated surfaces, medical equipment, or healthcare personnel [8]. Cross-transmission occurs when MDROs are transmitted from one patient to another via healthcare workers’ hands or shared medical devices. This is a common route of transmission in healthcare settings, especially when infection control practices are not strictly followed [8,9]. Invasive medical procedures such as the use of catheters or ventilators and surgical interventions have been identified as a contributing factor to the risk of healthcare-associated infection (HAI). This is because these procedures bypass the skin barrier as a body’s natural defense, providing a direct pathway for MDROs into sterile compartments to cause infections [10]. Inappropriate use of antibiotics contributes significantly to the emergence and selection of MDROs in the healthcare environment. Certain patient-related factors increase the risk of MDRO acquisition, including prolonged hospitalization, underlying chronic conditions, immunosuppression, and previous antibiotic use. These factors can compromise a patient’s ability to resist infections and make them more susceptible to acquired MDROs. Understanding these mechanisms is crucial for developing effective prevention and control strategies [10].

The key pathogens involved in HAIs according to the CDC’s reports are methicillin-resistant *Staphylococcus aureus* (MRSA), vancomycin-resistant *Enterococcus faecium* (VRE), carbapenem-resistant Enterobacterales (CRE), extended-spectrum beta-lactamase (ESBL)-producing Enterobacterales, multidrug-resistant *Pseudomonas aeruginosa*, multidrug-resistant *Acinetobacter baumannii*, *Clostridioides difficile*, and *Candida auris* (an emerging multidrug-resistant fungus that can cause severe systemic infections in hospitalized patients) [11].

Antimicrobial resistance is rising to alarming high levels in all parts of the world [7]. New resistance mechanisms are emerging and spreading globally, threatening our ability to treat communicable infectious diseases [7]. In May 2024, the WHO published the updated version of its Bacterial Priority Pathogens List, thus requiring the research and development of new molecules [12]. This list enumerates 15 families of bacteria most threatening to human health and assigns them to three categories: a critical group, a high group, and a medium group [12]. This work by the WHO is part of its efforts to combat growing antimicrobial resistance around the world [12]. It is a tool to ensure that research and development meet urgent public health needs.

According to the WHO bacterial priority pathogens list, the critical group of multidrug-resistant bacteria poses a particular threat in hospitals for patients who need surgery or whose care requires the use of devices such as respirators or vascular catheters [12]. It includes *Acinetobacter baumannii* producing a carbapenemase and various Enterobacterales resistant to carbapenems and/or third-generation cephalosporins [12]. They are often involved in lethal infections, such as blood stream infections, surgical site infections, and pneumonia. The high and medium priority groups include other increasingly resistant bacteria causing diseases such as gonorrhea or salmonella food poisoning, which are primarily acquired outside of medical care. Whereas priority 2 (high) consists of germs, e.g., *Pseudomonas aeruginosa* (carbapenem-resistant), *Enterococcus faecium* (vancomycin-resistant), *Staphylococcus aureus* (Methicillin-resistant), and *Neisseria gonorrhoeae* (resistant to third-generation cephalosporins or fluoroquinolones), piority 3 (medium) is made up of *Streptococcus pneumoniae* (macrolide-resistant), *Haemophilus influenzae* (ampicillin-resistant), and group A and B Streptococci (macrolide- or penicillin-resistant) [12].

The COVID-19 pandemic has had a profound impact on healthcare systems worldwide, radically altering clinical practices and infection dynamics. This disruption has raised concerns about the impact on HAIs particularly with regard to MDROs. The increased use of broad-spectrum antibiotics to treat secondary bacterial infections in COVID-19 patients exerted selective pressure on pathogens, potentially accelerating the development of resistance [13]. Previous studies worldwide highlighted an increase in the antibiotic resistance of various pathogens during COVID-19 [14,15,16,17,18,19,20], and a CDC analysis reported that hospital-onset infections with MDROs and related deaths increased by at least 15% from 2019 to 2020 among several pathogens [13].

Although many reports indicate an increase [21,22,23] in nosocomial infections, other studies show a decrease [24,25] in these infections during the COVID-19 pandemic. This is often explained by the fact that the pandemic led to some improvements in infection control practices: heightened awareness of pathogen transmission risks resulted in enhanced hand hygiene and more consistent use of personal protective equipment, and increased focus on cleaning and disinfection practices helped reduce the spread of certain pathogens. A study carried out in China concluded that these prevention and control measures for the COVID-19 pandemic have reduced the nosocomial infection rate in almost all departments, except intensive care units (ICUs) [26]. These mixed results reflect uncertainty about the effect of the COVID-19 pandemic on hospital-acquired infections [27].

In Germany, epidemiological data on this subject are still scarce. This study therefore offers a unique opportunity to fill a knowledge gap concerning the impact of COVID-19 on nosocomial infections caused by MDROs in a large university hospital in Germany. Targeted interventions can be derived by identifying specific risk factors and resistance trends to improve patient care and MDR-HAI management.

## 2. Materials and Methods

### 2.1. Study Design and Population

We conducted a cross-sectional monocentric and retrospective survey with a descriptive section and an analytic section during a six-month period from June to December 2024. On the basis of all inpatients of the University of Leipzig Medical Center (ULMC) before (2017–2019) and during (2021–2023) the COVID-19 pandemic (general population), we only included in the study population patients in whose selected sampling materials selected bacterial pathogens were detected.

Datasets were retrieved from the database of ULMC, including two institutes: the Institute for Medical Microbiology and Virology, for the microbiological datasets, and the Institute of Hygiene, Hospital Epidemiology and Environmental Medicine, for clinical and demographical data of the inpatients.

### 2.2. Outcome and Definitions

The outcome in this study was the acquisition of MDROs in defined clinical specimens in the healthcare setting as a surrogate for bacterial infections.

An infection was defined as the presence of one of the selected MDROs in one of the selected biological specimens from a patient hospitalized at the ULMC and detected during one year of the pre-COVID-19 period (2017–2019) or the COVID-19 period (2021–2023). According to the surveillance definition of the CDC’s National Healthcare Safety Network, an infection was classified as hospital-acquired when the MDRO acquisition time (time elapsed between the admission date and the MDRO detection date) was greater than or equal to 48 h (two days). In contrast to that, community-acquired infections are defined as manifesting on admission or within 48 h [28].

This study considered four biological materials that were routinely analyzed for the diagnosis of the most common nosocomial infections: urine, blood, intra-operative specimen, and bronchoalveolar lavage (BAL). We considered the following bacterial pathogens on the WHO list: carbapenem-resistant *Acinetobacter baumannii*, carbapenem-resistant *Pseudomonas aeruginosa*, carbapenem-resistant *Escherichia coli*, carbapenem-resistant *Klebsiella pneumoniae* (CRKP), carbapenem-resistant *Enterobacter cloacae (E. cloacae)*, extended-spectrum beta-lactamase (ESBL)-producing *Escherichia coli (E. coli)*, ESBL-producing *Klebsiella pneumoniae (K. pneumoniae)*, ESBL-producing *E. cloacae*, vancomycin-resistant *Enterococcus faecium* (VRE), and methicillin-resistant *Staphylococcus aureus* (MRSA).

### 2.3. Data Processing

Patients’ data were retrospectively surveyed from two existing databases at both institutes. Data were collected including demographical (identifier, sex, and age), clinical (admission date, admission unit, detection date of MDRO, and dismission date), and microbiological data (MDROs and their resistance profile). The two datasets were linked using the patient identifier. The data were then sorted and analyzed according to the type of biological material (urine, blood, intra-operative specimen, and BAL).

For the description of the study population, a case was defined as a patient identifier per year and per period (before and during COVID-19). During the analyses, a case of MDRO infection was defined using a unique patient identification code (patient-ID) by clinical material, reported MDRO, year, and period of detection (before and during COVID-19).

The interpretation of the susceptibility profile of each MDRO to its selected relevant antimicrobial agents (Table 1) was based on the breakpoint table from the guidelines of the European Committee on Antimicrobial Susceptibility Testing [29].

### 2.4. Data Analysis

The retrieved data were collected in Excel 2016 and analyzed by STATA 18.0 software. A Chi-square test was used for qualitative variables and Student’s test (*t*-test) for quantitative variables. For small sample sizes, a Yates’ continuity correction was used to adjust the Chi-square test, reducing the Chi-square value to correct the overestimation of significance. To study the factors associated with MDR-HAIs, we used logistic regression in univariable and multivariable analyses. The adjustment variables (those with *p* < 0.25 in the univariable analysis) were included in the multivariable model [30], and the “unit” variable was forced in the model (like HAIs’ potential risk factors reported in the literature). The data analysis also included the calculation of absolute proportions, mean, and odds ratio (OR) with their 95% confidence intervals (CI). For *p* < 0.05, the difference was considered statistically significant.

To investigate the impact of the COVID-19 period on risk factors associated with MDR HAIs, we used two logistic regression models: a baseline model without interaction terms to identify the main factors associated with MDR HAIs and a model with interaction terms to assess the effects specific to the COVID-19 period. The interaction terms were used to calculate specific odds ratios (ORs) before and during COVID-19 by combining the baseline ORs (before COVID-19) with the additional effect of the period. In other words, the OR during COVID-19 for each variable was calculated by multiplying the OR before COVID-19 by the additional OR corresponding to the interaction with the period. The use of models with interaction terms enabled us to quantify the additional effect of the COVID-19 period on the associations between factors and the acquisition of MDR infections.

The likelihood ratio test showed that the model with interactions significantly improved fit compared with the basic model, underlining the importance of including these interactions for accurate interpretation (*p* = 0.01).

### 2.5. Ethical Considerations

This study was approved by the Ethics Committee of Medical Research of the University of Leipzig (N° 267/24-ck, 27 August 2024) in accordance with the Declaration of Helsinki from 1975 (revision 2013) and the International Conference on Harmonization/Committee for Proprietary Medicinal Products “Good Clinical Practice” guidelines.

### 2.6. Flow Chart

**Figure 1:**
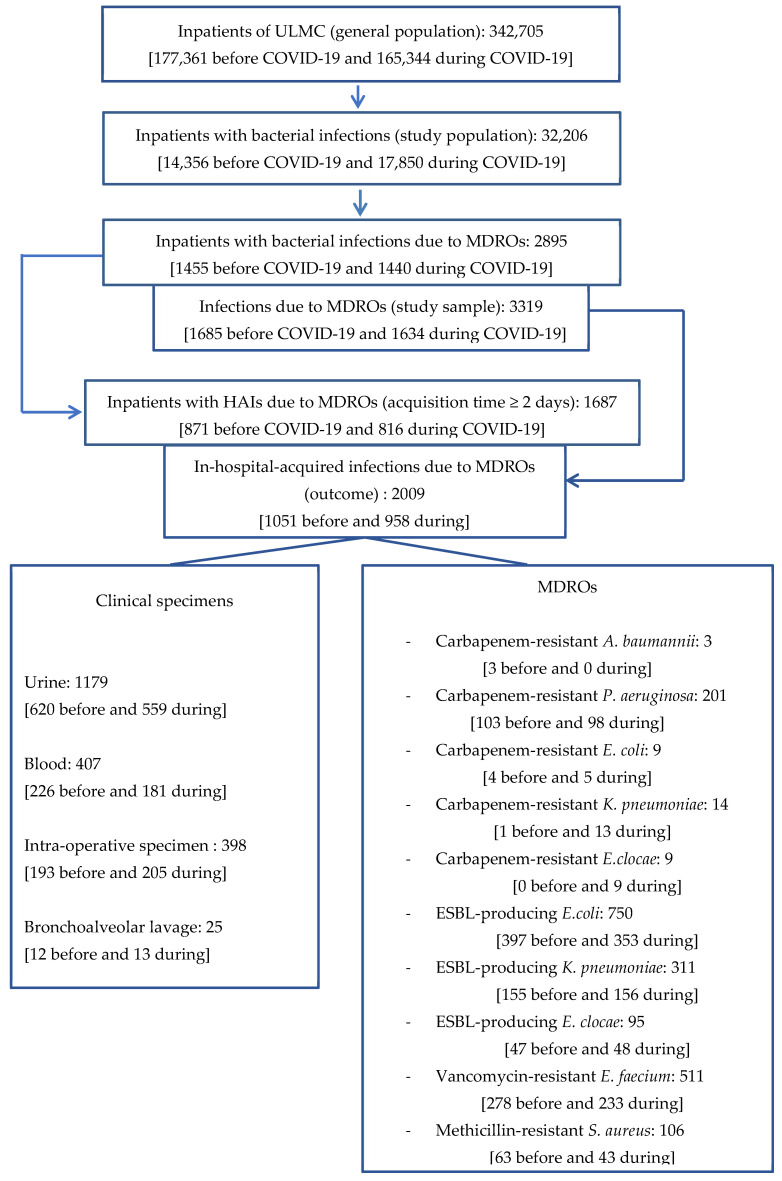
Flow chart.

For illustration of the methodological process, the flow chart highlights the key steps of our approach (Figure 1).

## 3. Results

### 3.1. Description of the General Population

We surveyed the data of 342,705 hospitalized patients at the ULMC within two time periods: 2017–2019 (before COVID-19) and 2021–2023 (during COVID-19). Table 2 summarizes the characteristics of the general population.

It appears from the characteristics of the general population that the proportion of hospitalized patients was lower during the COVID-19 pandemic (48.25% (165,344/342,705)) compared to before (51.75% (177,361/342,705)). Similar observations were made for females and adults (*p* < 0.001). In contrast, increases in the proportions of males and elderly patients (*p* < 0.001) and for an in-hospital stay of 15–30 days (*p* = 0.01) were observed.

Taking together the data from both periods, there were more male (M) than female (F) patients from 2017 to 2023 [172,330/342,705 (50.25%) versus 167,249/342,705 (48.80%)], and the overall sex ratio (M/F) was 1.03. The most represented age group was adults, 164,070/342,705 (47.87%), followed by elderly patients, 121,233/342,705 (35.38%). About 74.01% (255,001/342,705) of the patients had a length of stay in hospital within 7 days; 93.38% of the patients (320,012/342,705) were admitted to a normal care unit, while only 6.62% (22,693/342,705) of them were admitted to an intensive care unit.

### 3.2. Description of the Study Population

Data of 32,206 hospitalized patients with bacterial infections were retrieved from the inpatients’ database of the ULMC in two time periods: 2017–2019 (before COVID-19) and 2021–2023 (during COVID-19). Table 3 summarizes the general characteristics of the study population.

The number of hospitalized patients with bacterial infections was higher during the COVID-19 pandemic period compared to before [55.42% (17,850/32,206) during vs. 44.58% (14,356/32,206) before, *p* < 0.001]. The same trend was observed in the proportions of the elderly group (*p* = 0.05) and for short stays and stays in a normal care unit (*p* < 0.001). In contrast, decreases in the proportions of males, infants, very long stays, and stays in an intensive care unit were observed (*p* < 0.05).

There were more males (M) than females (F) with bacterial infections in both periods (53.02% versus 46.96% before and 51.90% versus 47.61% during). Overall, the sex ratio (M/F) was 1.11. The most represented age group was elderly patients (>65 years) (55.07%), followed by adults (39.10%). About 33.09% of patients had a length of stay in hospital within 7 days, and 66.91% had a stay of more than 7 days accordingly, with 78.63% of them being admitted to a normal care unit and 21.37% being admitted to an intensive care unit.

### 3.3. Prevalence of Bacterial Infections in Inpatients Before (2017–2019) and During (2021–2023) COVID-19

We calculated and compared the prevalence of bacterial infections for both periods in the general population (see Table 2).

**Figure 2:**
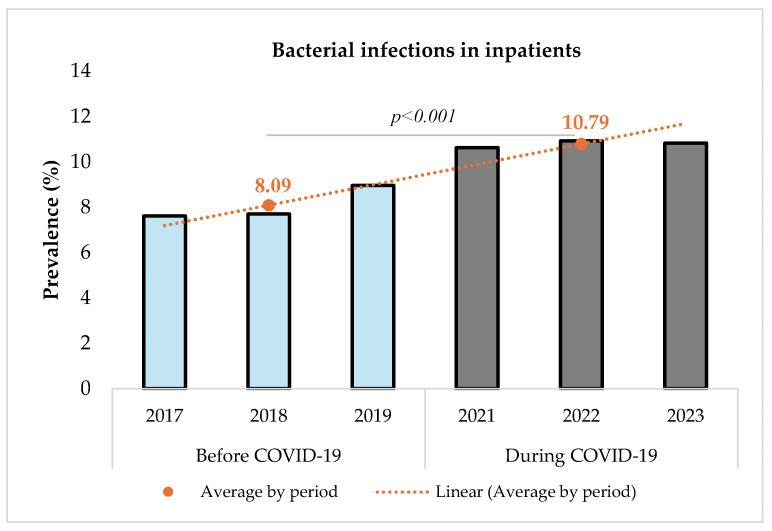
Prevalence of bacterial infections among inpatients before and during COVID-19.

Figure 2 shows that the prevalence of bacterial infections increased significantly from 8.09% (14,356/177,361) before to 10.79% (17,850/165,344) during the pandemic (*p* < 0.001). The comparison was performed using a Chi-square test with a significance level set at 5%. The Chi-square test resulted in a value of 733.12 (*p* < 0.001), indicating a statistically significant difference between the two periods.

### 3.4. Infections Due to MDROs in Inpatients Before (2017–2019) and During (2021–2023) COVID-19

**Figure 3:**
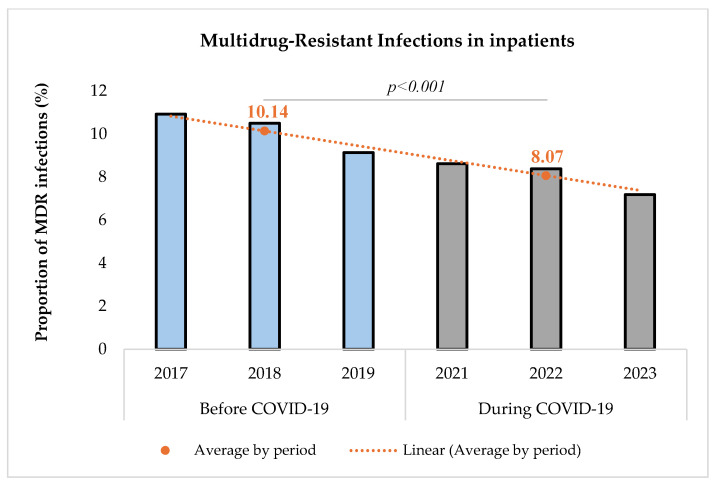
Proportion of multidrug-resistant infections before and during COVID-19.

The proportion of MDR infections among the study group of inpatients with a bacterial infection was determined and compared between the two periods (Figure 3).

The proportion of MDR infections among infected inpatients decreased significantly during the COVID-19 pandemic [(10.14% (1455/14,356) before vs. 8.07% (1440/17,850) during, *p* < 0.001)]. The comparison was performed using a Chi-square test with a significance level set at 5%. The Chi-square test resulted in a value of 41.34, indicating a statistically significant difference between the two periods.

### 3.5. Multidrug-Resistant Healthcare-Acquired Infections (HAIs) in Inpatients Before (2017–2019) and During (2021–2023) COVID-19

The proportion of in-hospital-acquired MDR infections was determined and compared between the two periods.

**Figure 4:**
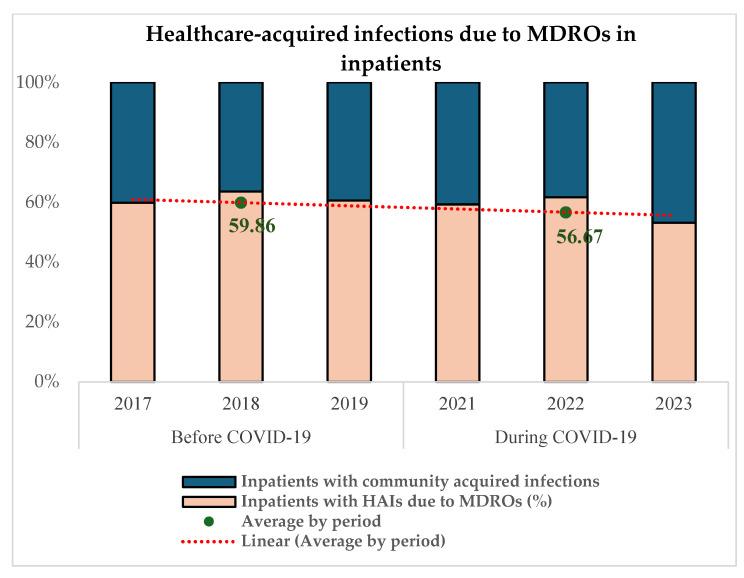
Proportion of in-hospital-acquired MDR infections before and during COVID-19.

Figure 4 reveals that 59.86% (871/1455) of MDRO infections among infected inpatients were acquired in the healthcare setting in the pre-COVID-19 period and 56.67% (816/1440) during COVID-19 (*p* = 0.06).

### 3.6. Distribution of Healthcare-Acquired MDRO

**Figure 5:**
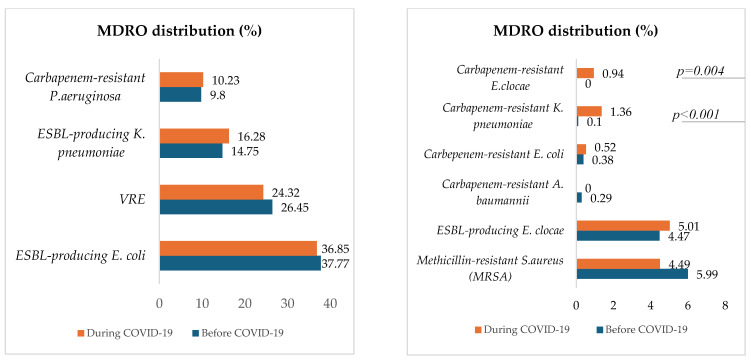
Proportions of in-hospital-acquired MDROs before and during COVID-19.

Figure 5 describes and compares the distribution of MDROs for both periods.

Except for carbapenem-resistant *K. pneumoniae*, which significantly increased during COVID-19 (*p* < 0.001), the proportions of selected hospital-acquired MDROs, were approximately the same between the two periods. The most represented MDRO was ESBL-producing *E. coli*, followed by vancomycin-resistant *E. faecium* (VRE), ESBL-producing *K. pneumoniae*, carbapenem-resistant *P. aeruginosa*, Methicillin-resistant *S. aureus* (MRSA), and ESBL-producing *E. clocae*. Carbapenem-resistant *E. clocae* was isolated only during the COVID-19 period.

### 3.7. Distribution of MDR-HAIs in Clinical Specimens Before and During the COVID-19 Pandemic

**Figure 6:**
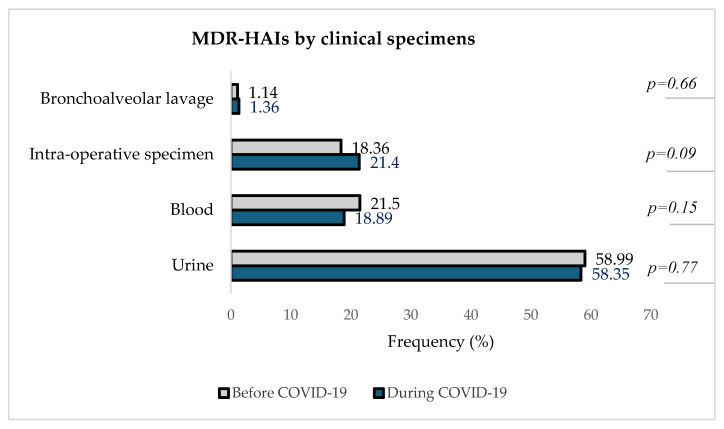
Distribution of MDR-HAIs by clinical specimens before and during theCOVID-19 pandemic.

Figure 6 describes the distribution of detected MDROs in different clinical specimens in the two periods.

Figure 6 indicates that urine was the clinical specimen with the highest proportion of MDR-HAIs both before and during the pandemic, with 58.99% (620/1051) pre-COVID-19 and 58.35% (559/958) during COVID-19, followed by blood samples [21.50% (226/1051) before vs. 18.89% (181/958) during]. In intra-operative specimens, the number of in-hospital-acquired MDROs increased from 18.36% (1931/1051) pre-COVID-19 to 21.40% (205/958) during COVID-19. The isolation of MDROs from bronchoalveolar lavage remained the least represented, about 1% in both periods.

### 3.8. Antibiotic Susceptibility Profile of Healthcare-Acquired MDRO

The antibiotic susceptibility profile for each in-hospital-acquired MDRO was assessed before and during the COVID-19 pandemic.

#### 3.8.1. ESBL-Producing Enterobacterales: *E. coli*, *K. pneumoniae*, *E. clocae*

**Figure 7:**
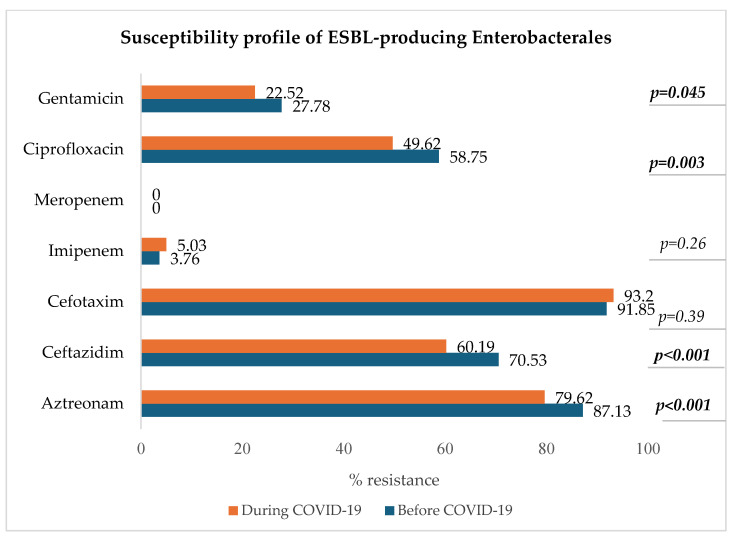
Antibiotic susceptibility profile of ESBL-producing Enterobacterales.

Figure 7 shows a decrease in resistance to ciprofloxacin, ceftazidim, aztreonam, and gentamicin in the group of ESBL-producing Enterobacterales (*p* < 0.05).

#### 3.8.2. Carbapenem-Resistant Enterobacterales: *E. coli*, *K. pneumoniae*, *E. clocae*

**Figure 8:**
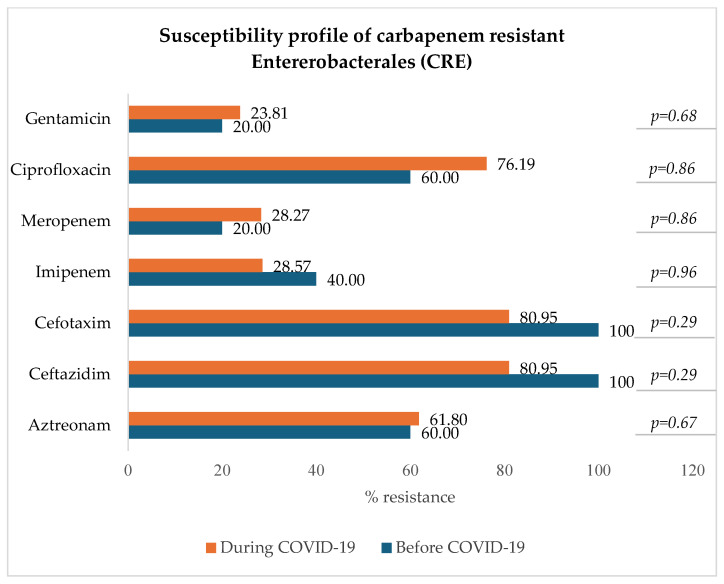
Antibiotic susceptibility profile of CRE.

Figure 8 indicates that among carbapenem-resistant Enterobacterales (resistant to either imipenem, meropenem, or both), there was a slight and not statistically significant decrease in resistance to imipenem (from 40.00% to 28.57%) and a slight increase in resistance to meropenem (from 20.00% to 28.27%), ciprofloxacin (*p* = 0.86), and gentamicin (*p* = 0.68).

#### 3.8.3. Carbapenem-Resistant *Acinetobacter baumannii* (CRAB)

Three isolates of carbapenem-resistant *Acinetobacter baumannii* were identified at the ULMC exclusively in the pre-COVID-19 period. All isolates were tested to be resistant to both ciprofloxacin and gentamicin.

#### 3.8.4. Carbapenem-Resistant *Pseudomonas aeruginosa* (CRPA)

**Figure 9:**
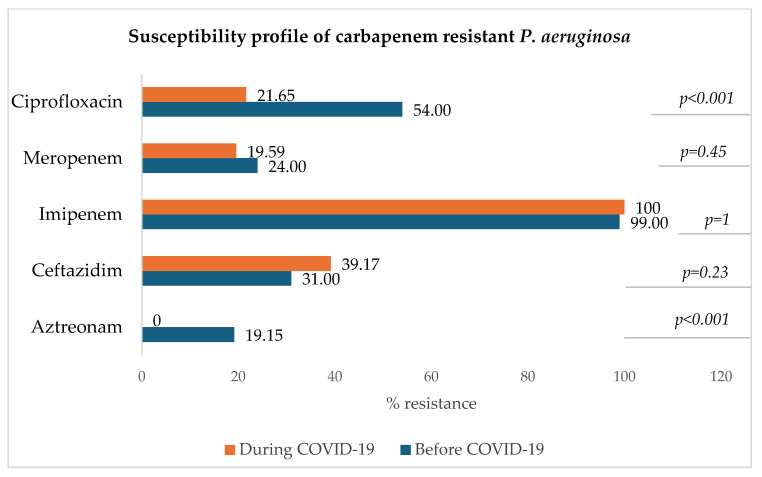
Antibiotic susceptibility profile of carbapenem-resistant *P. aeruginosa*.

Figure 9 shows that for carbapenem-resistant *P. aeruginosa*, the resistance rate to imipenem remained very high during both periods (100% and 99%) and that of meropenem has slightly decreased during the COVID-19 era in comparison to the pre-COVID-19 period. A significant decrease in the resistance to ciprofloxacin was observed (*p* < 0.001).

#### 3.8.5. Vancomycin-Resistant *Enterococcus faecium* (VRE)

Linezolid resistance of VRE did not differ significantly between the two periods (*p* = 0.09) with very high susceptibility rates in both periods: 100% before COVID-19 and 98.13% during COVID-19.

#### 3.8.6. Methicillin-Resistant *Staphylococcus aureus* (MRSA)

The susceptibility of MRSA to linezolid, daptomycin, and vancomycin was 100% before and during the COVID-19 pandemic.

### 3.9. Factors Associated with the In-Hospital Acquisition of Infections Due to MDROs (MDR-HAIs)

#### 3.9.1. Univariable Analysis with Interaction Terms (Variable*Period COVID-19)

In the univariable analysis, Table 4 shows the following:

- Compared to adults (18–64 years), the risk of in-hospital-acquired infections due to MDROs was 0.34 times lower in infants under one year of age (*p* = 0.019) before the COVID-19 pandemic. This was non-significantly lower in children (*p* = 0.12) and the elderly group (*p* = 0.37). During COVID-19, there was a significant decrease in the odds ratio (OR) of the association between MDR-HAIs and the elderly group (OR = 0.66 vs 0.91 before, *p* = 0.03).
- Compared to a length of stay (LOS) < 8 days, the risk of MDR-HAIs tended to increase (trend *p* < 0.001) in both periods for longer LOSs. Before the pandemic, the OR increased significantly from 2.32 (*p* < 0.001) for medium stays (8–14 days) to 9.45 (*p* < 0.001) for long stays (15–30 days) and to 33.60 (*p* < 0.001) for very long stays (> 30 days) compared to short stays. During COVID-19, there was also an increase in OR between all these types of stay and MDR HAIs (respectively, 2.32 to 2.69, 9.45 to 12.66, and 33.60 to 45.70). But, the additional effect of this increase was not significant.
- Compared to normal care, the risk of in-hospital-acquired infections due to MDROs was 1.33 times higher in intensive care units (*p* = 0.03) before the pandemic. During COVID-19, although not significant (*p* = 0.67), the OR for this association increased to 1.44.

#### 3.9.2. Multivariable Analysis (Logistic Regression with Interaction Terms)

We performed a model with interaction terms to assess the effects specific to the COVID-19 period on the relation between factors and the in-hospital acquisition of MDR infections (Table 5).

This study analyzed the relationship between factors such as age, length of stay, and levels of care, and the risk of MDR-HAIs across the pre-COVID-19 and COVID-19 periods. To understand how these associations changed with the onset of the pandemic, we added interaction terms to the multivariable analysis. This approach allowed for the additional effect of the COVID-19 period on the risk of MDR-HAIs to be demonstrated. Table 5 illustrates the following results:

- During COVID-19, there was a significant decrease in the risk of MDR-HAIs among the children (OR = 1.55 to 0.31, *p* = 0.003) and elderly (OR = 1.17 to 0.78, *p* = 0.03) groups. Compared to an LOS < 8 days, the risk of MDR-HAIs tended to increase (trend *p* < 0.001) in both periods for longer LOSs. Before the pandemic, the LOS was a risk factor of MDR-HAIs. We therefore noted a significant increase in the risk to 2.31 [1.63–3.18] (*p* < 0.001) for medium stays, to 9.48 [6.87–13.36] (*p* < 0.001) for long stays, and to 34.27 [23.62–49.73] (*p* < 0.001) for very long stays compared to short stays. During COVID-19, the odds ratios (OR) for MDR-HAIs increased for medium stays (OR = 2.68), long stays (OR = 12.80), and very long stays (OR = 48.52), but these increases were not statistically significant. 3.9.3. Multivariable Analysis (Logistic Regression Without Interaction Terms)

We also performed a model without interaction terms to assess the factors associated with the in-hospital acquisition of MDR infections over the entire study period (2017–2023) (see Table 6).

Without the interaction terms, Table 6 shows the following:

- During the COVID-19 pandemic, the risk of in-hospital acquisition of infections due to MDROs in infected inpatients at ULMC decreased to 0.82 [0.67–0.99] compared to the pre-pandemic period (*p* = 0.047).
- The length of stay was a risk factor to MDR HAIs, with an increased risk of 2.47 [1.87–3.27] for medium stays, 10.57 [8.02–13.94] for long stays, and 34.73 [25.37–47.53] for very long stays compared to the short stays (*p* < 0.001). Risk increased with length of stay (trend *p* < 0.001).

## 4. Discussion

Healthcare systems worldwide face significant challenges due to MDR-HAIs, which lead to higher morbidity, mortality, and healthcare expenses [4,31]. The WHO has noted that the burden of MDR-HAIs was notably impacted by the dramatic changes brought about by the COVID-19 pandemic [32]. Some studies have been conducted early on during the pandemic and analyzed the changes caused by the lockdown in the first half of the year 2020, while other studies focused on patients treated for COVID-19 disease. To our knowledge, this is the first study that investigates the dynamics and epidemiology of in-hospital-acquired infections due to MDROs among inpatients at a German university hospital in a three-year period before and during the COVID-19 pandemic. We refrained from analyzing the year 2020 because we wanted to examine the longer-term impact of the pandemic and because of the results of the study published by Geffers et al. [33] that showed that the pandemic had no impact on the rates of nosocomial infections acquired in German ICUs during the early pandemic.

The number of hospitalized patients was slightly lower during the pandemic (48.25%) compared to the period before 2020 (51.75%). This trend was consistent across other characteristics, such as the proportion of females and adult patients and for a length of stay of 15–30 days (*p* < 0.001). This decline is in line with the findings of Kapsner’s study [34], which reported that admission rates to university hospitals in Germany fell significantly after the national COVID-19 lockdown in the first half of 2020. Our results show that this decrease in hospitalizations continued in the later years of the pandemic.

Bacterial infections are a significant concern in healthcare settings due to their potential to cause severe health complications, prolonged hospital stays, and increased medical expenses. The most frequently reported types of HAIs are respiratory tract infections, surgical site infections, urinary tract infections, blood stream infections, and infections with *Clostridioides difficile* [35]. In the study population (hospitalized patients with bacterial infections in ULMC from 2017 to 2023), there were more males (M) than females (F) from 2017 to 2023 (52.40% versus 47.32%), and the overall sex ratio (M/F) was 1.11. The most represented age group was elderly patients (>65 years) (55.07%), followed by adults (39.10%). These demographics align with trends reported in the literature. For instance, a study on COVID-19 hospitalizations in the U.S. found that adults aged 65 years and older accounted for a significant proportion of hospitalizations, reflecting their vulnerability to severe health outcomes [4,36].

Some studies have reported an increase in antimicrobial resistance (AMR) during the COVID-19 pandemic, mainly due to increased antibiotic use in patients with COVID-19 in ICUs, but the data are highly heterogeneous [14,37]. Langford et al. [38] reported in a systematic review that the COVID-19 pandemic was not associated with a change in the incidence density or proportion of MRSA or VRE cases but that a non-statistically significant increase was noted for resistant Gram-negative organisms.

We found in our study that the proportion of infections due to MDROs among inpatients decreased significantly during the pandemic (10.14% before vs. 8.07% during, *p* < 0.001) although the number of bacterial infections increased (8.09% vs. 10.79%). Our study focuses on a broader population of hospitalized patients, not limited to those infected with COVID-19, which may explain the observed decrease in the proportion due to MDROs.

More than 50% (59.86% before and 56.67% during) of multidrug-resistant infections in hospitalized patients had their onset in the hospital in both periods (Figure 4).

Urine was the most represented source of MDR-HAIs, followed by blood and intra-operative specimens and BAL. These findings are consistent with global trends in healthcare-associated infections, as reported by the CDC, which emphasized the prevalence of HAIs and the need for robust infection control measures [39].

With the exception of carbapenem-resistant *K. pneumoniae* (CRKP), which increased significantly during COVID-19 (*p* < 0.001), the proportions of selected hospital-acquired MDROs were approximately the same between the two periods. Possible reasons for this finding are that there were no changes in antimicrobial stewardship practices or isolation precautions before and during the pandemic, but that the cohort during the pandemic included some patients from Ukraine, a region where multidrug-resistant bacteria, including strains of CRKP, have been identified and reported, especially among war-injured patients [40,41]. To prevent the spread of these resistant strains in healthcare settings, the European Centre for Disease Prevention and Control (ECDC) has, since March 2022, recommended pre-emptive isolation and screening for multidrug-resistant bacteria, particularly carbapenem-resistant Enterobacterales, for patients transferred from hospitals in Ukraine or with a history of hospital admission in Ukraine in the past 12 months [42]. The significant increase in CRKP during the COVID-19 pandemic aligns with recent studies and reports, highlighting CRKP as a major concern. For instance, a study published in *PLOS Pathogens* emphasized the emergence of KPC-2 (*Klebsiella pneumoniae* carbapenemase-2)-producing CRKP as a predominant strain, underscoring the need for new therapeutic strategies [43].

The most common MDRO was ESBL-producing *E. coli*, followed by vancomycin-resistant *E. faecium* (VRE), ESBL-producing *K. pneumoniae*, carbapenem-resistant *P. aeruginosa*, methicillin-resistant *S. aureus* (MRSA), and ESBL-producing *E. clocae*. Additionally, the resistance to ciprofloxacin of ESBL-producing strains significantly decreased during the pandemic. Another study conducted by Ilmavirta et al. highlighted a decrease in the proportion of ESBL-producing *E. coli* in urine and blood isolates during the pandemic, also reporting that the proportion of fluoroquinolone-resistant isolates among ESBL-producing *E. coli* decreased only slightly during 2019–2022 [44].

All these findings align with the systematic review and meta-analysis performed by Kariyawasam et al. from 2019 to 2021 which indicated that while some studies reported an increase in resistance to certain antibiotics, others observed a decrease [14]. These mixed trends in antibiotic resistance could be attributed to changes in prescribing practices during the pandemic.

A univariable analysis of our study population revealed the previously described risk of MDR-HAIs in ICUs, which was 1.37 compared to normal care units (*p* = 0.01) before the pandemic. During COVID-19, although not significant (*p* = 0.77), the OR for this association increased to 1.33.

Consistent with the results of the European point prevalence survey of healthcare-associated infections [39], the risk of MDR-HAIs increased significantly with increasing length of stay, with no significant change over the course of the pandemic.

Interestingly, in the multivariable regression logistic model without interaction terms, the risk of acquired multidrug-resistant infections during the COVID-19 pandemic was significantly reduced, by 18% (OR = 0.82 [0.67–0.99]), compared to before the COVID-19 pandemic onset (*p* = 0.047). This reduction may be attributed to enhance infection control measures implemented during the pandemic, such as improved hand hygiene, increased use of personal protective equipment (PPE), and stricter isolation protocols.

These findings underscore the importance of continued vigilance in infection control and antibiotic stewardship, which is essential to improve preparedness for managing infections due to MDROs in hospitals, particularly during crises such as a pandemic, and to ensure that healthcare systems can respond effectively to future public health emergencies.

Our study has some strengths but also some limitations. The strength lies in the fact that all inpatients of the entire hospital were included to calculate the prevalence of defined bacterial infections. The study population included all patients with cultural bacterial pathogen detection from defined biological materials collected for the diagnosis of bacteremia (blood culture), urinary tract infection (urine), pneumonia (BAL), or infections related to surgery (intraoperatively collected material). This selection allowed us to focus on the most clinically significant and most frequently occurring nosocomial infections. Our study analyzed the prevalence of bacterial infections and the proportion of MDR-HAIs in a 3-year period before the COVID-19 pandemic (2017–2019) compared to the period from 2021 to 2023, i.e., during the pandemic.

Concerning the limitations, firstly, this study was conducted at a single medical center; therefore, the results may not be applicable to other hospitals with different patient populations, resources, and infection control practices. Secondly, the retrospective nature of this study might introduce biases related to data collection and accuracy, as it relies on existing records. Finally, some potential confounding factors such as preexisting MDRO colonization, patient comorbidities, or differences in healthcare worker practices that were not measured or controlled in the course of this study could also have influenced the results.

For future directions, it would be beneficial to conduct a multicenter study to enhance external validity by recruiting a more diverse and representative patient sample. Expanding the scope of determinants to include factors such as comorbidities, care practices, antibiotic use, and environmental and institutional factors would also be valuable. Additionally, focusing on less frequent and less critical multidrug-resistant bacteria could improve early detection and prevention strategies, leading to more comprehensive infection control measures and better preparedness for future public health crises. Conducting the same analysis on patients with infections caused by non-multidrug-resistant bacteria could help to determine if the same trends are observed between the two periods, supporting the explanation of improved infection prevention methods during the pandemic.

## 5. Conclusions

To sum up, this study highlights the impact of the COVID-19 pandemic on in-hospital-acquired infections due to MDROs at a German university hospital. Despite a slight decrease in hospital admissions during the pandemic, the number of bacterial infections increased. The proportion of infections due to MDROs among inpatients decreased significantly during the pandemic and the risk of MDR-HAIs remained closely linked to the length of hospital stay. Our findings underscore the need to analyze epidemiological data at the facility level in order to evaluate the effectiveness of infection control practices even during unprecedented health crises like the COVID-19 pandemic.

## Figures and Tables

**Table 1:** MDRO and selected antimicrobial agents.

Selected MDRO	Selected Antimicrobial Agents
ESBL-producing or carbapenem-resistant Enterobacterales: *E. coli, K. pneumoniae, E. cloacae*	aztreonam, ceftazidim, cefotaxim, imipenem, meropenem, ciprofloxacin, gentamicin
Carbapenem-resistant *A. baumannii*	imipenem, meropenem, ciprofloxacin, gentamicin
Carbapenem-resistant *P. aeruginosa*	aztreonam, ceftazidim, imipenem, meropenem, ciprofloxacin
VRE	vancomycin, linezolid
MRSA	vancomycin, daptomycin, linezolid

**Table 2:** Characteristics of the general population.

Characteristics		Before COVID-19 n = 177,361 (51.75%)		During COVID-19 n = 165,344 (48.25%)		Total n = 342,705		*p*-Value ^a^
		n	%	n	%	n	%	
**Sex**								
	Male	88,608	49.95	83,722	50.64	172,330	50.25	**<0.001**
	Female	88,232	49.75	79,017	47.79	167,249	48.80	**<0.001**
	Unknown	0	0.00	6	0.004	6	0.002	0.03
	Missing	521	0.30	2599	1.57	3120	0.91	<0.001
**Age (years)**								
	*	48		50		49		
	Infants (<1)	12,314	6.94	11,280	6.82	23,594	6.88	0.16
	Children (1–17)	17,43	9.83	16,375	9.90	33,808	9.87	0.46
	Adults (18–64)	87,986	49.61	76,084	46.02	164,070	47.87	**<0.001**
	Elderly (65+)	59,628	33.62	61,605	37.26	121,233	35.38	**<0.001**
**Stay (days)**								
	*	7		7		7		
	Short stay (1–7)	132,206	74.54	122,795	74.27	255,001	74.41	0.07
	Medium stay (8–14)	24,464	13.79	22,852	13.82	47,316	13.81	0.8
	Long stay (15–30)	13,766	7.76	13,212	8.00	26,978	7.87	**0.01**
	Very long stay (>30)	6925	3.90	6470	3.91	13,395	3.91	0.9
	Missing	0	0.00	15	0.01	15	0.004	<0.001
**Unit of care**								
	Normal care unit	165,493	93.31	154,519	93.45	320,012	93.38	0.09
	Intensive care unit	11,868	6.69	10,825	6.55	22,693	6.62	0.09

* Mean, ^a^ Chi2 test, *p* < 0.05: significant difference between the proportions before and during COVID-19.

**Table 3:** General characteristics of the study population.

Characteristics		Before COVID-19 n = 14,356 (44.58%)		During COVID-19 n = 17,850 (55.42%)		Total N = 32,206		*p*-Value ^a^
		n	%	n	%	n	%	
**Sex**								
	Male	7612	53.02	9265	51.90	16,877	52.40	**0.046**
	Female	6741	46.96	8498	47.61	15,239	47.32	0.24
	Missing	3	0.02	87	0.49	90	0.28	<0.001
**Age (years)**								
	*	62		62		62		
	Infants (<1)	344	2.40	318	1.78	662	2.06	**<0.001**
	Children (1–17)	523	3.64	695	3.89	1218	3.78	0.24
	Adults (18–64)	5669	39.49	6922	38.78	12,591	39.10	0.2
	Elderly (≥65)	7820	54.47	9915	55.55	17,735	55.07	**0.05**
**Stay (days)**								
	*	20		18		19		
	Short stay (1–7)	4545	31.66	6111	34.24	10,656	33.09	**<0.001**
	Medium stay (8–14)	3302	23.00	4344	24.34	7646	23.74	0.005
	Long stay (15–30)	3717	25.89	4577	25.64	8294	25.75	0.61
	Very long stay (>30)	2792	19.45	2814	15.76	5606	17.41	**<0.001**
	Missing	0	0.00	4	0.02	4	0.01	
**Unit of care**								
	Normal care unit	11,167	77.79	14,155	79.30	25,322	78.63	**<0.001**
	Intensive care unit	3189	22.21	3695	20.70	6884	21.37	**<0.001**

* Mean, ^a^ Chi2 test, *p* < 0.05: significant difference between the proportions before and during COVID-19.

**Table 4:** Univariable analysis of associated factors (inpatient characteristics) for in-hospital acquisition of MDRO infections.

Variables		In-Hospital Acquisition of MDRO									
		Before COVID-19					During COVID-19				
		n	No (%)	Yes (%)	OR ^c^	*p*-Value ^c^	n	No (%)	Yes (%)	OR ^c^	*p*-Value ^c^*
**Sex ^a^**											
	Male	863	323 37.43	540 62.57	Ref		841	332 39.48	509 60.52	Ref	
	Female	822	311 37.83	511 62.17	0.98	0.86	788	343 43.53	445 56.47	0.84	0.29
	Missing	-	-	-	1	-	4	0 0.00	4 100.0	1	-
**Age (years) ^b^**		1685 63 *	634 63 *	1051 63 *		0.51 ^b^	1633 61 *	675 62 *	958 61 *		0.35 ^b^
**Group of age ^a^**											
	Adults	698	250 35.82	448 64.18	Ref		740	262 35.41	478 64.59	Ref	
	Infants	21	13 81.90	8 38.10	0.34	**0.019**	17	7 41.18	10 58.83	0.77	0.22
	Children	44	21 47.73	23 52.27	0.61	0.12	54	36 66.67	18 33.33	0.27	0.06
	Elderly	922	350 37.96	572 62.04	0.91	0.37	822	370 45.01	452 54.99	0.66	**0.03**
**Length of stay (days) ^b^**		1685 32 *	634 13 *	1051 43 *		**<0.001** ^b^	1632 30 *	674 12 *	958 42 *		**<0.001** ^b^
**Type of stay ^a^**						<0.001 **					
	Short stay	348	275 79.02	73 20.98	Ref		361	299 82.83	62 17.17	Ref	
	Medium stay	273	169 61.90	104 38.1	2.32	**<0.001**	344	221 64.24	123 35.76	2.69	0.57
	Long stay	449	128 28.51	321 71.49	9.45	**<0.001**	362	100 27.62	262 72.38	12.66	0.24
	Very long stay	615	62 10.08	553 89.92	33.60	**<0.001**	565	54 9.56	511 90.44	45.70	0.26
**Unit ^a^**											
	Normal care unit	1354	527 38.92	827 61.08	Ref		1310	564 43.05	746 56.95	Ref	
	Intensive care Unit	331	107 32.33	224 67.67	1.33	**0.03**	323	111 34.37	212 65.63	1.44	0.67

* Mean, ^a^ Chi2 test, ^b^ Student’s test, ^c^ logistic regression, ** trend test, ^c^* *p*-value of the additional effect introduced by the interaction between the variable and the COVID-19 period.

**Table 5:** Factors associated with in-hospital-acquired MDR infections before and during the COVID-19 pandemic in a multivariable analysis with the interaction terms.

		In-Hospital-Acquired MDRO (MDR HAIs)						
		Before COVID-19			During COVID-19			
		OR ^c^	CI (95%) ^c^	*p*-Value ^c^	Additional OR ^c^*	CI (95%) ^c^*	OR ^c^	*p*-Value ^c^**
**Group of age**								
	Adults	Ref						
	Infants	0.70	[0.24–2.70]	0.52	1.14	[0.22–5.85]	0.80	0.88
	Children	1.55	[0.75–3.20]	0.23	0.20	[0.07–0.58]	0.31	**0.003**
	Elderly	1.17	[0.91–1.50]	0.23	0.67	[0.47–0.96]	0.78	**0.03**
**Type of stay**				<0.001 **				
	Short stay	Ref						
	Medium stay	2.31	[1.62–3.31]	**<0.001**	1.16	[0.70–1.92]	2.68	0.55
	Long stay	9.48	[6.87–13.36]	**<0.001**	1.35	[0.83–2.21]	12.80	0.23
	Very long stay	34.27	[23.62–49.73]	**<0.001**	46.61	[0.79–2.35]	48.52	0.27
**Unit**								
	Normal care unit	Ref						
	Intensive care unit	1.14	[0.83–1.55]	0.42	0.66	[0.42–1.03]	0.75	0.06

** Trend test, ^c^ logistic regression, ^c^* effect of interaction (variable*period COVID-19), ^c^** *p*-value of the additional effect introduced by the interaction between the variable and the COVID-19 period.

**Table 6:** Overview of the factors associated with in-hospital-acquired infections due to MDROs over the entire study period.

Variables		In-Hospital-Acquired MDR Infections				
		No n (%)	Yes n (%)	Adjusted OR	IC (95%)	*p*-Value
**COVID-19 Era**						
	Before COVID-19	634 (37.63)	1051 (62.37)	Ref		
	During COVID-19	675 (41.33)	958 (58.67)	0.82	[0.67–0.99]	**0.047**
**Type of stay**			<0.001 **			
	Short stay	574 (80.96)	135 (19.04)	Ref		
	Medium stay	390 (63.21)	227 (36.79)	2.47	[1.87–3.27]	**<0.001**
	Long stay	228 (28.11)	583 (71.89)	10.57	[8.02–13.94]	**<0.001**
	Very long stay	116(9.83)	1064 (90.17)	34.73	[25.37–47.53]	**<0.001**

** Trend test.

## Data Availability

The datasets used and/or analyzed during the current study are available from the corresponding author on reasonable request.
